# Diversity of Antibiotic Biosynthesis Gene-possessing Rhizospheric Fluorescent Pseudomonads in Japan and Their Biocontrol Efficacy

**DOI:** 10.1264/jsme2.ME19155

**Published:** 2020-04-07

**Authors:** Nobutaka Someya, Masaharu Kubota, Kasumi Takeuchi, Yusuke Unno, Ryohei Sakuraoka, Tomohiro Morohoshi

**Affiliations:** 1 Institute of Vegetable and Floriculture Science, National Agriculture and Food Research Organization (NARO), 3–1–1 Kannondai, Tsukuba, Ibaraki 305–8519, Japan; 2 Institute of Agrobiological Sciences, NARO, Kannondai, Tsukuba, Ibaraki 305–8602, Japan; 3 Institute for Environmental Sciences, 1–7 Ienomae, Obuchi, Rokkasho, Kamikita, Aomori 039–3212, Japan; 4 Department of Innovation Systems Engineering, Graduate School of Engineering, Utsunomiya University, 7–1–2 Yoto, Utsunomiya 321–8585, Japan; 5 Department of Material and Environmental Chemistry, Graduate School of Engineering, Utsunomiya University, 7–1–2 Yoto, Utsunomiya 321–8585, Japan

**Keywords:** fluorescent pseudomonads, biocontrol, 2,4-diacetylphloroglucinol, phenazine, *Rhizoctonia solani*

## Abstract

More than 3,000 isolates of fluorescent pseudomonads have been collected from plant roots in Japan and screened for the presence of antibiotic-synthesizing genes. In total, 927 hydrogen cyanide (HCN)-, 47 2,4-diacetylphloroglucinol (PHL)-, 6 pyoluteorin (PLT)-, 14 pyrrolnitrin (PRN)-, and 8 phenazine (PHZ)-producing isolates have been detected. A cluster analysis (≥99% identity) identified 10 operational taxonomic units (OTUs) in antibiotic biosynthesis gene-possessing pseudomonads. OTU HLR (PHL, PLT, and PRN) contained four antibiotics: HCN, PHL, PLT, and PRN, while OTU RZ (PRN and PHZ) contained three: HCN, PRN, and PHZ. OTU H1, H2, H3, H4, H5, H6, and H7 (PHL1-7) contained two antibiotics: HCN and PHL, while OTU H8 (PHL8) contained one: PHL. Isolates belonging to OTU HLR and RZ suppressed damping-off disease in cabbage seedlings caused by *Rhizoctonia solani*. Effective strains belonging to OTU HLR and RZ were related to *Pseudomonas protegens* and *Pseudomonas chlororaphis*, respectively. Antibiotic biosynthesis gene-possessing fluorescent pseudomonads are distributed among different geographical sites in Japan and plant species.

The genus *Pseudomonas* is one of the most ubiquitous bacteria in multiple environments, including soil and water, and is frequently found in animals and phytospheres (Hirano and Upper, 2000; Palleroni and Moore, 2004; Peix *et al.*, 2009). Although most *Pseudomonas* species are saprophytic and avirulent, some are causal agents for both plant and animal diseases (Hirano and Upper, 2000; D’Argenio, 2004). On the other hand, some *Pseudomonas* species are plant-beneficial microbes that are utilized as biofungicides or biofertilizers (Haas and Défago, 2005; Höfte and Altier, 2010; Anderson and Kim, 2018). Fluorescent pseudomonads are a specific group of *Pseudomonas* species that produce an extracellular, water-soluble, yellow-green pigment that fluoresces under UV irradiation. Fluorescent pseudomonads have been isolated from a number of samples, and many exhibit biocontrol activities and protect host plants via multiple mechanisms, including the production of antibiotics, induction of host resistance, and various benefits to nutrient or niche competition. These biocontrol fluorescent pseudomonads produce antimicrobial secondary metabolites, including hydrogen cyanide (HCN), 2,4-diacetylphloroglucinol (PHL), pyoluteorin (PLT), pyrrolnitrin (PRN), and phenazines (PHZ) (Morrisey *et al.*, 2004; Gross and Loper, 2009). Each of these compounds displays potent biocontrol efficacy against phytopathogens within their respective antimicrobial spectrums (Howell and Stipanovic, 1979; Howell and Stipanovic, 1980, Voisard *et al.*, 1989; He *et al.*, 2004). Some biocontrol isolates contain multiple antibiotics and control a wide range of plant diseases caused by various phytopathogens. However, since the relationship between antibiotic production and biocontrol efficacy has mainly been investigated in specific model strains of fluorescent pseudomonads, the diversity of fluorescent pseudomonads isolated from different environments and the distribution of antibiotic genes within these isolates remain unclear.

In Japan, many fluorescent pseudomonads have been isolated and demonstrated to have potent biocontrol efficacy (Oshiman *et al.*, 1996; Tazawa *et al.*, 2000; Zhang *et al.*, 2000; He *et al.*, 2004). Although the biocontrol properties (including antibiotic production) of some fluorescent pseudomonads isolated in Japan have been characterized, most have not been properly investigated for biocontrol efficacy. Furthermore, since most isolates were identified as *Pseudomonas fluorescens* or *P. putida*, the diversity of fluorescent pseudomonads found in Japan has not yet been investigated in detail. On the other hand, some plant-protecting isolates of *P. fluorescens* were recently re-identified as a novel species (Ramette *et al.*, 2011). However, the distribution and phenotypic characteristics of plant-protecting *Pseudomonas* species in Japan remain unclear. In the present study, we attempted to investigate the distribution of antibiotic biosynthesis gene-possessing fluorescent pseudomonads in agronomic fields in Japan and test the biocontrol efficacy of isolates using a basic pathosystem technique.

## Materials and Methods

### Plant sampling and isolation of fluorescent pseudomonads

The surfaces of individual 1-g root samples collected from various fields in Japan were washed with 9‍ ‍mL of sterile 15‍ ‍mM phosphate buffer (pH 7.0) and then sonicated with an ultrasonic washer (USM-1; SND) at 42 kHz for 1‍ ‍min to release microbial cells from the roots. Serial dilutions were cultivated on King’s B (KB) medium agar containing 50‍ ‍μg mL^–1^ cycloheximide and incubated at 25°C in the dark for 3 d. After cultivation, fluorescent pseudomonads were identified after ultraviolet irradiation at 365 nm using an ultraviolet lamp (MODEL UVGL-58; UVP).

### PCR amplification of antibiotic-synthesizing genes using specific primers

Isolated bacterial strains were inoculated into 2‍ ‍mL Luria-Bertani (LB) liquid medium (Sigma-Aldrich Japan) and incubated at 25°C for 24 h on a reciprocal shaker (140 rpm). Bacterial cells were collected by centrifugation, and total bacterial genomic DNA was extracted using the DNeasy blood and tissue kit (Qiagen) according to the manufacturer’s instructions. Specific primer sets for the amplification of antibiotic-synthesizing genes (*hcnAB* and *hcnBC* for HCN; *phlD* for PHL; *pltB* and *pltC* for PLT; *prnC* and *prnD* for PRN; and *phzCD* and *phzF* for PHZ) were described previously (Someya *et al.*, 2013). PCR products were separated by electrophoresis performed on 1.5% agarose gels, stained with ethidium bromide, and visualized with a transilluminator (FAS-III; Toyobo).

### Sequence analysis of 16S rRNA genes

16S rRNA genes were amplified by PCR with Premix Taq (Takara Bio) from genomic DNA obtained from bacterial isolates. The primers used were as follows: 27F (5′-AGAGTTTGATCMTGGCTCAG-3′) and 1525R (5′-AAGGAGGTGWTCCARCC-3′). The thermal cycling program began with an initial denaturation at 94°C for 3‍ ‍min, followed by 30 cycles at 94°C for 30‍ ‍s, 55°C for 30‍ ‍s, and 72°C for 1‍ ‍min, with a final extension at 72°C for 10‍ ‍min. Sequencing was conducted on 16S rRNA genes using the 27F, 1525R, f1L (5′-GTATTACCGCGGCTGCTGG-3′), f2L (5′-CCAGCAGCCGCGGTAATAG-3′), and 926f (5′-AAACTCAAAGGAATTGACGG-3′) primers from the Takara Dragon Genomic Center (Takara Bio). Sequences were placed in a taxonomic hierarchy using the Classifier in Ribosomal Database Project (RDP) II (Cole *et al.*, 2003), and sequences from non-*Pseudomonas* species were eliminated. A cluster analysis was performed via a previously described method (Someya *et al.*, 2012). The operational taxonomic units (OTUs) in the cluster analysis were defined by 99% sequence identity. Representative sequences of OTUs were aligned using CLUSTAL X and used to build a phylogenetic tree by the neighbor-joining (NJ) method (Saitou and Nei, 1987) with type strains of known species. The topology of the constructed tree was evaluated by a bootstrap analysis with 1,000 replicates (Felsenstein, 1997). The trees were constructed using TreeView software (Page, 1996).

### Identification and quantification of quorum-sensing signal molecules

*N*-acyl homoserine lactone (AHL), a quorum-sensing signal, may be detected by the AHL reporter strains, *Chromobacterium violaceum* CV026 and VIR24 (Someya *et al.*, 2009). Isolates were inoculated onto LB agar medium, and the two AHL reporter strains, CV026 and VIR24, were inoculated onto the same medium adjacent to isolate colonies. After an incubation at 30°C for 2‍ ‍d, AHL-producing activity was detected by measuring the production of purple pigment by AHL reporter strains.

### Biocontrol test

To assess the effectiveness of isolates against phytopathogens, a cabbage-*R. solani* pathosystem was used. Damping-off caused by *R. solani* is a serious disease in a number of vegetables. A mycelial disc (5‍ ‍mm in diameter) of *R. solani* MAFF726551 was cut from colonies grown on potato sucrose agar (PSA) and placed on a water agar (WA) plate (55‍ ‍mm in diameter). Plates were incubated at 28°C for 7‍ ‍d in the dark. Bacterial strains were incubated on KB medium agar at 28°C in the dark for 3 d. Cabbage (*Brassica oleracea* var. *capitate* L. ‘Shosyu’) was used as the test plant in the present study, and seeds were surface-sterilized in 70% ethanol for 30‍ ‍s and in 2.5% sodium hypochlorite for 1‍ ‍min, then rinsed three times with sterile distilled water. Seeds were then inoculated with bacterial cells and placed on WA plates. Bacterial cells were detected at approximately 5×10^8^ to 1×10^9^ colony-forming units (CFU) seed^–1^, and plates were incubated at 28°C for 24 h in the dark. In comparisons, the anti-microbial agent fluazinam (Ishihara Sangyo) was used as a chemical control.

Eight bacterized seeds were inoculated onto WA plates containing *R. solani* and overlaid with sterilized vermiculite (Takamura). Three plates were tested with each treatment. Plates were transferred into a sterilized plant box (Asahi Glass), and plant boxes were incubated at 28°C for 14‍ ‍d in a multi thermo incubator (MTI-204; Tokyo Rikakikai). Disease severity was evaluated according to the following damage index: 0=no symptoms, 1=discoloration of the hypocotyl, 2=damping-off, and 3=collapse of seedlings. Disease severity was calculated by the following formula: ([0×*n*_0_+1×*n*_1_+2×*n*_2_+3×*n*_3_]/24). Each experiment was replicated three times. The disease severity of the control treatment was calculated and set to 100% in order to represent the disease incidence for the control. The disease incidence of the bacterial treatment was compared with the control treatment using Dunnett’s test. Statistical analyses were performed using BellCurve software (Social Survey Research Information).

### Nucleotide sequence accession number

The nucleotide sequences of the 16S rRNA genes have been deposited in the DDBJ/ENA/GenBank database under accession numbers LC420166–LC420220.

## Results

### Detection of antibiotic biosynthesis genes in fluorescent pseudomonads isolated from plant roots

Approximately 500 root samples from 88 distinct plant species, including crops and weeds, were collected from 135 fields located in 19 different prefectures (Hokkaido, Yamagata, Niigata, Fukushima, Ibaraki, Tochigi, Gunma, Saitama, Tokyo, Kanagawa, Nagano, Aichi, Shiga, Kyoto, Osaka, Hiroshima, Fukuoka, Nagasaki, and Kagoshima) in Japan. Five colonies that showed fluorescence on KB agar under UV light were isolated from each sample. In total, 3,115 fluorescent pseudomonads were isolated. The results of 16S rRNA gene sequencing revealed that all isolates belonged to the genus *Pseudomonas*.

The presence of antibiotic biosynthesis genes in the DNA of isolates was assessed via PCR with specific primers. Antibiotic biosynthesis genes were not detected in 2,186 isolates. Nine hundred and twenty-seven isolates only possessed the HCN synthesis gene, *hcn*, and two isolates only possessed the PHL synthesis gene, *phl*. Thirty-eight isolates possessed both *hcn* and *phl*, eight possessed, *hcn*, *prn*, and *phz*, and only five possessed all four genes, (*hcn*, *phl*, *plt*, and *prn*).

### Phylogenetic diversity of fluorescent pseudomonads positive for antibiotic biosynthesis genes

**Fig. 1.**
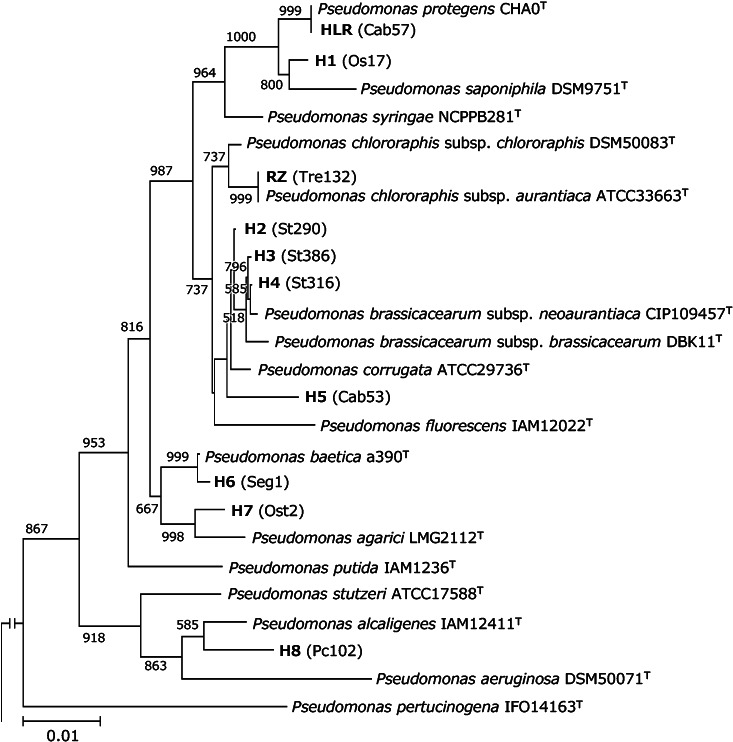
Phylogenetic tree of 16S rRNA genes based on operational taxonomic units (representative isolate) for antibiotic-producing fluorescent pseudomonads. The tree was constructed using the neighbor-joining method. The scale represents 0.01 substitutions per site. The numbers at the nodes represent the proportions of 1,000 bootstrap resamplings, and values of <500 are not shown.

A phylogenetic analysis was performed, excluding isolates possessing only the HCN biosynthesis gene. The cluster analysis (>99% identity) revealed the presence of 10 OTUs among isolates possessing the antibiotic biosynthesis genes *phl*, *plt*, *prn*, and *phz* (Table 1). Strains together with their origin and OTU are indicated in Table S1. In brief, OTU HLR possessed four antibiotic biosynthesis genes: *hcn*, *phl*, *prn*, and *plt*. However, two isolates categorized as OTU HLR were each missing one of these genes, either *prn* or *plt*, respectively (Table S1). 16S rRNA gene sequencing revealed that OTU HLR was closely related to *P. protegens* (Fig. 1 and S1). OTU RZ possessed three antibiotic biosynthesis genes: *hcn*, *prn*, and *phz*, and was shown to be related to *P. chlororaphis* (Fig. 1 and S2). OTU H2 to H7 possessed two antibiotic biosynthesis genes: *hcn* and *phl*. OTU H2 and H3–H5 were related to *P. corrugata* and *P. brassicacearum*, respectively (Fig. 1 and S3). OTU H6, which shows the distinct amplicon patterns of *phlD* observed using the primer sets phl2a and phl2b (data not shown), was related to *P. baetica* (Fig. 1 and S4). OTU H7 was related to *P. agarici* (Fig. 1 and S4), and OTU H8 possessed the *phl* gene, but not the *hcn* gene, and was shown to be related to *P. alcaligenes* (Fig. 1 and S4).

### Evaluation of quorum-sensing signal molecule production

Previous studies showed that the biosynthesis of PHZ and PRN was regulated by the quorum sensing system via AHL (Selin *et al.*, 2012; Morohoshi *et al.*, 2013). Thus, we measured AHL production by all isolates using AHL reporter strains. The results obtained showed that eight isolates from OTU RZ produced AHL with a short acyl chain (Table 1).

### Evaluation of biocontrol efficacy and relationship between the biosynthesis of antibiotics

**Fig. 2.**
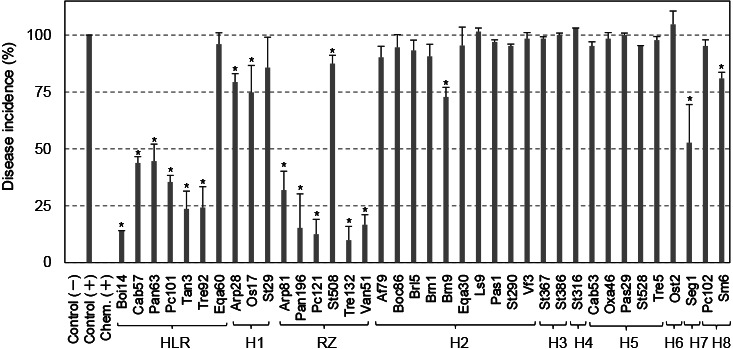
Effects of fluorescent pseudomonad isolates on cabbage damping-off caused by *Rhizoctonia solani*. As a comparison, a healthy plant (Control [–]), disease control (Control [+]), and chemical control (Chem. [+]) are also shown. Treatment groups that differ significantly from the disease control are indicated by an asterisk (*P*<0.05). Error bars indicate SD.

To assess the contribution of antibiotic biosynthesis genes to biocontrol capacity, we used a cabbage-*R. solani* pathosystem. Isolates belonging to OTU HLR and RZ exhibited strong antifungal and biocontrol activities (Fig. 2). Most isolates belonging OTU H1–H8 did not exhibit any significant plant-protecting activity in bioassays using the cabbage-*R. solani* pathosystem (Fig. 2). However, some isolates, Brn9, Seg1, and Sm6, belonging OTU H2, H7, and H8 showed low biocontrol efficacy.

## Discussion

Some fluorescent pseudomonads protect host plants from plant pathogens via antibiotic production (Voisard *et al.*, 1989; Oshiman *et al.*, 1996; Tazawa *et al.*, 2000; Morrisey *et al.*, 2004; Someya *et al.*, 2007; Mazurier *et al.*, 2009; Takeuchi *et al.*, 2015; Ma *et al.*, 2016; Nandi *et al.*, 2017). Antibiotic-producing pseudomonads are isolated and utilized as biopesticides worldwide (Haas and Défago, 2005; Höfte and Altier, 2010; Anderson and Kim, 2018).

In the present study, all isolates used in bioassays possessed the PHL gene, *phl*. Most isolates possessed the HCN biosynthesis gene *hcn*, with the exception of OTU H8. Previous studies reported that PHL and HCN both play important roles in the biocontrol of plant pathogens by fluorescent pseudomonads (Voisard *et al.*, 1989; Schippers *et al.*, 1990; Nandi *et al.*, 2017). However, only a few isolates of OTU H1, H2, H3, H4, H5, H6, H7, and H8 showed biocontrol efficacy, while many did not. Therefore, the presence of *phl* or *phl* and *hcn* does not appear to be related to biocontrol efficacy based on the results of the bioassays performed in the present study (Fig. 2). PRN biosynthesis genes were detected in OTU HLR and RZ (Table 1). PLT or PHZ biosynthesis genes were only detected in OTU HLR and RZ, respectively. OTU HLR and RZ, which showed high biocontrol efficacy, both possessed the PRN biosynthesis gene. PRN is an effective antifungal metabolite against *R. solani* (Howell and Stipanovic, 1979). On the other hand, PLT has been shown to inhibit *Pythium*, but not *Rhizoctonia* (Howell and Stipanovic, 1980). PHZ is also an effective antibiotic against phytopathogens, including *R. solani* (Kapsalis *et al.*, 2008; Mazurier *et al.*, 2009; Morohoshi *et al.*, 2013). These findings indicate that the PRN biosynthesis gene is a primary factor, and PHZ is a secondary factor with biocontrol efficacy in the present study. However, not all *prn*-containing isolates exhibit the same biocontrol activity, and plant-protecting activity may depend on isolate- and context-dependent antibiotic productivity in the rhizosphere. All *prn*- or *phz*-possessing isolates also included other antibiotic biosynthesis genes, such as *phl*, *plt*, and *hrn*. These antibiotics may function as synergistic factors in effective isolates.

Multiple antibiotic-possessing isolates were found to be effective against *R. solani* in the present bioassay. However, it currently remains unclear whether these isolates protect plants via the production of multiple antibiotics under the rhizosphere environment because previous studies reported that PHL and PLT mutually inhibited the other’s production in *P. protegens* (Schnider-Keel *et al.*, 2000; Brodhagen *et al.*, 2004; Kidarsa *et al.*, 2011). Therefore, the biosynthesis of PLT is inhibited under conditions in which isolates produce PHL, such as in the present study. For example, when all *phl*-possessing isolates were incubated on diluted nutrient agar plus yeast extract (Duffy and Défago, 1999), PHL was detected in all isolates using thin layer chromatography assays, whereas PLT was not (data not shown). Furthermore, PRN production in *P. chlororaphis* was shown to be regulated by the quorum sensing system via AHL signaling molecules (Selin *et al.*, 2012). Isolates belonging to OTU RZ produced AHL, whereas those belonging to OTU HLR did not (Table 1). The genome sequence of St508 from OTU RZ was analyzed, and showed the presence of three independent quorum-sensing systems (Morohoshi *et al.*, 2017). In contrast, the genome sequences of Cab57 from OTU HLR did not contain AHL synthase (Takeuchi *et al.*, 2014). These results suggest that the biosynthesis of the same antibiotic, such as PRN, is regulated by different mechanisms in the genus *Pseudomonas*. These results also indicate that the biosynthesis of other antibiotics, including PHL and PLT, is not regulated by AHL-mediated quorum sensing (Corbell and Loper, 1995; Haas and Défago, 2005). The biosynthesis of pyrrolnitrin is considered to be important; however, environmental conditions and interactions with other antibiotics may also affect biocontrol efficacy. Therefore, further analyses are needed to identify the primary factor affecting biocontrol efficacy in each isolate and their productive environmental conditions.

Antibiotic-producing pseudomonads are enriched in disease-suppressive soils (Landa *et al.*, 2006; Mazurier *et al.*, 2009). The frequency of isolation of antibiotic-producing fluorescent pseudomonads in the rhizosphere differs based on geological location, host plants, farm management, and various environmental conditions (Raaijmakers *et al.*, 1997). For example, *phl*-possessing pseudomonads were detected in naturally suppressive soils as more than 10% of fluorescent pseudomonads, and in non-suppressive soils at less than 0.1% of fluorescent pseudomonads (Raaijmakers *et al.*, 1997). Similarly, *phz*-possessing fluorescent pseudomonads were detected in disease-suppressive soils at approximately 10^3^ to 10^4^ CFU g^–1^ in roots, but were below the detection limit of 10^2^ CFU g^–1^ in roots from disease-conducive soils (Mazurier *et al.*, 2009). In the present study, *phl*-possessing isolates were detected in plant roots at approximately 1.5%, and *phz*-possessing isolates at approximately 0.3%, of all isolates. Complex mechanisms may be contributing to the density of specific-antibiotic biosynthesis gene-possessing pseudomonads affecting soil suppressiveness against phytopathogens. Disease-suppressive pseudomonad species and others appear to co-exist under the same conditions.

Effective isolates were species that belonged to two species, *P. protegens* and *P. chlororaphis*. These isolates were isolated from different samples based on geographical location and plant species, including crops and weeds. The present results showed that the two species are widely distributed in Japan (Table S1). A previous study reported that Pseudomonadaceae related to *P. brassicacearum*, *P. kilonensis*, and *P. thivervalensis* were more abundant in disease-suppressive than -conductive soil (Mendes *et al.*, 2011). However, not all *phl*-containing isolates related to the above species, such as OTU H2, H3, H4, and H5, exhibit effective biocontrol activity. Many antibiotic biosynthesis gene-possessing isolates were eventually isolated; however, promising isolates were limited to two species, *P. protegens* and *P. chlororaphis*.

*P. protegens* is a plant-protecting species that produces the antibiotics PHL and PLT (Ramette *et al.*, 2011). OTU H1, which possessed two antibiotic biosynthesis genes, *hcn* and *phl*, was partially related to OTU HLR, but showed greater similarities to *P. saponiphila* than to *P. protegens* (Fig. S1). We previously obtained the whole-genome sequences of representative isolates from these two OTUs: Cab57 from OTU HLR and Os17 and St29 from OTU H1 (Takeuchi *et al.*, 2014; 2015). In that study, Cab57 was identified as *P. protegens*. Although Os17 and St29 from OTU H1 were related to *P. saponiphila*, which is a decomposer of xenobiotic compounds (Lang *et al.*, 2010), it currently remains unclear whether this species exhibits biocontrol activity against plant pathogens. Further analyses of this species and related isolates are needed.

*P. chlororaphis* is a plant-protecting species that produces the antibiotics PHZ and PRN (Someya and Morohoshi, 2019). It has been divided into four subspecies: *P. chlororaphis* subsp. *chlororaphis*, *P. chlororaphis* subsp. *aurantiaca*, *P. chlororaphis* subsp. *aureofaciens*, and *P. chlororaphis* subsp. *piscium* (Peix *et al.*, 2007). Although the eight isolates mapped to OTU RZ were closely related to *P. chlororaphis* subsp. *aurantiaca* and *aureofaciens*, a phylogenetic analysis using only 16S rRNA gene sequences did not distinguish them at the subspecies level (Fig. S2). A genome sequence analysis revealed that most *P. chlororaphis* strains had the same three antibiotic biosynthesis genes: *hcn*, *prn*, and *phz* (Loper *et al.*, 2012; Shen *et al.*, 2013). We previously demonstrated that the complete genome sequences of St508 from OTU RZ also contained *hcn*, *prn*, and *phz* (Morohoshi *et al.*, 2017). On the other hand, *P. chlororaphis* strain UFB2 possessed the *hcn* and *phl* genes instead of the *phz* and *prn* genes (Deng *et al.*, 2015), while *P. chlororaphis* strain PCL1606 possessed the *hcn* and *prn* genes, but not the *phz* gene (Calderón *et al.*, 2015). Therefore, the antibiotic biosynthesis genes of isolates of *P. chlororaphis* are considered to be very diverse.

Since isolates possessing antibiotic genes were found at diverse collection sites and plant species in the present study, no clear relationship was observed between antibiotic-producing pseudomonads and the origins of samples. Effective isolates belonging to OTU HLR and RZ were obtained at different sites and host plants. These OTUs were closely related to *P. protegens* and *P. chlororaphis* species. Therefore, it does not appear to be coincidental that effective isolates belonged to these two species. Both species are distributed in various environments (Someya *et al.*, 2017; Someya and Morohoshi, 2019). The genomes of both species are large in the genus *Pseudomonas*, and produce various secondary metabolites (Takeuchi *et al.*, 2014; Someya and Morohoshi, 2019). These large genomes may confer an environmental fitness advantage on these species, which are considered to have the potential to adapt to various environmental conditions. However, difficulties are associated with isolating and selecting effective isolates as plant-protecting bacteria under natural conditions. Selective isolation or enrichment methods in agricultural fields for both species may lead to efficient biological control strategies.

Fluorescent pseudomonad isolates that are plant-protective and produce multiple antibiotics are regarded as subgroups of *P. fluorescens* (Howell and Stipanovic, 1979, 1980; Voisard *et al.*, 1989; Tazawa *et al.*, 2000; Zhang *et al.*, 2000; Morrisey *et al.*, 2004; Someya *et al.*, 2007). However, many novel species have been proposed, and the genus *Pseudomonas* was recently reclassified (Gomila *et al.*, 2015; Tran *et al.*, 2017; Peix *et al.*, 2018). Therefore, some isolates from plant-protecting *Pseudomonas* have been re-identified as novel species, such as *P. protegens*, *P. brassicacearum*, and *P. synxantha* (Ramette *et al.*, 2011). The present results also demonstrated that the majority of isolates belonging to the two species of fluorescent pseudomonads, *P. protegens* and *P. chlororaphis*, were effective for cabbage damping-off in the present study. Furthermore, some isolates collected in the present study revealed that the novel plant-protecting species of pseudomonads were also widely distributed in Japan, and some isolates may be novel species with unknown abilities and ecological profiles. A phylogenetic analysis and increased understanding of their potential will be important for utilizing plant-protecting bacteria as biopesticides in agriculture.

In conclusion, plant-protecting fluorescent pseudomonads are widely distributed under natural conditions in Japan; however, the dominance of beneficial species under agricultural conditions using artificial methods has not yet been established. We anticipate the development of indigenous fluorescent pseudomonads as useful and effective biocontrol agents in sustainable agriculture after the further elucidation of their plant-protecting mechanisms. A more detailed understanding and development of the processing method of selected isolates will be necessary for their utilization under agricultural conditions.

## Citation

Someya, N., Kubota, M., Takeuchi, K., Unno, Y., Sakuraoka, R., and Morohoshi, T. (2020) Diversity of Antibiotic Biosynthesis Gene-possessing Rhizospheric Fluorescent Pseudomonads in Japan and Their Biocontrol Efficacy. *Microbes Environ ***35**: ME19155.

https://doi.org/10.1264/jsme2.ME19155

## Supplementary Material

Supplementary Material

## Figures and Tables

**Table 1.** Phylogenetic distribution of operational taxonomic units (OTUs) of antibiotic-producing fluorescent pseudomonads isolated from plant roots in various locations across Japan

OTU	Isolates	Antibiotics^a^					AHLs^b^	Closest known species^c^	Acc. No.	Identity (%)
		HCN	PHL	PHZ	PLT	PRN				
HLR	7	+	+	−	+	+	−	*Pseudomonas protegens*	NR_114749	100
H1	7	+	+	−	−	−	−	*Pseudomonas sesami*	NR_149822	99
RZ	8	+	−	+	−	+	+	*Pseudomonas chlororaphis*	NR_43935	99
H2	18	+	+	−	−	−	−	*Pseudomonas corrugata*	NR_117826	99
H3	2	+	+	−	−	−	−	*Pseudomonas brassicacearum*	NR_116299	99
H4	1	+	+	−	−	−	−	*Pseudomonas brassicacearum*	NR_116299	99
H5	7	+	+	−	−	−	−	*Pseudomonas gessardii*	NR_024928	99
H6	2	+	+	−	−	−	−	*Pseudomonas baetica*	NR_116899	99
H7	1	+	+	−	−	−	−	*Pseudomonas agarici*	NR_115608	99
H8	2	−	+	−	−	−	−	*Pseudomonas alcaligenes*	NR_113646	99
Total	55									

^a^ The presence of the antibiotic biosynthesis genes, hydrogen cyanide (HCN), 2,4-diacetylphloroglucinol (PHL), phenazine (PHZ), pyoluteorin (PLT), and pyrrolnitrin (PRN). ^b^ The production of *N*-acyl-homoserine lactones (AHLs). ^c^ The results of pair-wise BLAST between a representative sequence and its closest type strain.
