# Supplementary material for: Diversity of Antibiotic Biosynthesis Gene-possessing Rhizospheric Fluorescent Pseudomonads in Japan and Their Biocontrol Efficacy
Source: Microbes Environ. 2020 Apr 7;35(2):ME19155. doi: 10.1264/jsme2.ME19155 (PMC7308580; doi:10.1264/jsme2.ME19155)
Supplement: Supplementary file 1 — Supplementary Material [file 35_19155_s1.pdf]

Table S1. Antibiotic-biosynthesis genes-posseing fluorescent pseudomonads screened in this study

OTU	Strain	Host	Collection site	Antibiotic-biosynthesis gene				
				hcn	*phl*	*phz*	*plt*	*prn*
HLR	Boi14	*Brassica oleracea* var. *italica*	Koga, Ibaraki, Japan	+	+	−	+	−
HLR	Cab57	*Capsella bursa-pastoris* (L.) Medik.	Shikaoi, Hokkaido, Japan	+	+	−	+	+
HLR	Eqa60	*Equisetum arvense* L.	Monbetsu, Hokkaido, Japan	+	+	−	−	+
HLR	Pan63	*Poa annua* L.	Ryugasaki, Ibaraki, Japan	+	+	−	+	+
HLR	Pc101	*Phaseolus coccineus* L.	Tsukuba, Ibaraki, Japan	+	+	−	+	+
HLR	Tan3	*Tylophora aristolochioides* Miq.	Fujimi, Nagano, Japan	+	+	−	+	+
HLR	Tre92	*Trifolium repens* L.	Takahagi, Ibaraki, Japan	+	+	−	+	+
H1	Arp28	*Artemisia princeps* Pampan.	Hitachi, Ibaraki, Japan	+	+	−	−	−
H1	Os16	*Oryza sativa* L.	Hitachi, Ibaraki, Japan	+	+	−	−	−
H1	Os17	*Oryza sativa* L.	Hitachi, Ibaraki, Japan	+	+	−	−	−
H1	Os18	*Oryza sativa* L.	Hitachi, Ibaraki, Japan	+	+	−	−	−
H1	Os19	*Oryza sativa* L.	Hitachi, Ibaraki, Japan	+	+	−	−	−
H1	Os20	*Oryza sativa* L.	Hitachi, Ibaraki, Japan	+	+	−	−	−
H1	St29	*Solanum tuberosum* L.	Ibaraki, Ibaraki, Japan	+	+	−	−	−
H2	Af79	*Allium fistulosum* L.	Takahagi, Ibaraki, Japan	+	+	−	−	−
H2	Boc86	*Brassica oleracea* var. *capitata* L.	Hitachiota, Ibaraki, Japan	+	+	−	−	−
H2	Brl5	*Brassica rapa* var. *laciniifolia*	Hitachiota, Ibaraki, Japan	+	+	−	−	−
H2	Brn1	*Brassica rapa* var. *nippo-oleifera*	Hitachiota, Ibaraki, Japan	+	+	−	−	−
H2	Brn2	*Brassica rapa* var. *nippo-oleifera*	Hitachiota, Ibaraki, Japan	+	+	−	−	−
H2	Brn3	*Brassica rapa* var. *nippo-oleifera*	Hitachiota, Ibaraki, Japan	+	+	−	−	−
H2	Brn5	*Brassica rapa* var. *nippo-oleifera*	Hitachiota, Ibaraki, Japan	+	+	−	−	−
H2	Brn7	*Brassica rapa* var. *nippo-oleifera*	Hitachiota, Ibaraki, Japan	+	+	−	−	−
H2	Brn8	*Brassica rapa* var. *nippo-oleifera*	Hitachiota, Ibaraki, Japan	+	+	−	−	−
H2	Brn9	*Brassica rapa* var. *nippo-oleifera*	Hitachiota, Ibaraki, Japan	+	+	−	−	−
H2	Brn10	*Brassica rapa* var. *nippo-oleifera*	Hitachiota, Ibaraki, Japan	+	+	−	−	−
H2	Eqa30	*Equisetum arvense* L.	Tsukuba, Ibaraki, Japan	+	+	−	−	−
H2	Ls9	*Lactuca sativa* L.	Tsukuba, Ibaraki, Japan	+	+	−	−	−
H2	Pas1	*Plantago asiatica*	Fujimi, Nagano, Japan	+	+	−	−	−
H2	St290	*Solanum tuberosum* L.	Atsugi, Kanagawa, Japan	+	+	−	−	−
H2	St292	*Solanum tuberosum* L.	Atsugi, Kanagawa, Japan	+	+	−	−	−
H2	St293	*Solanum tuberosum* L.	Atsugi, Kanagawa, Japan	+	+	−	−	−
H2	Vf3	*Vicia faba* L.	Hitachiota, Ibaraki, Japan	+	+	−	−	−
H3	St367	*Solanum tuberosum* L.	Kiyosato, Hokkaido, Japan	+	+	−	−	−
H3	St386	*Solanum tuberosum* L.	Honbetsu, Hokkaido, Japan	+	+	−	−	−
H4	St316	*Solanum tuberosum* L.	Nakasatsunai, Hokkaido, Japan	+	+	−	−	−
H5	Cab52	*Capsella bursa-pastoris* (L.) Medik.	Honbetsu, Hokkaido, Japan	+	+	−	−	−
H5	Cab53	*Capsella bursa-pastoris* (L.) Medik.	Honbetsu, Hokkaido, Japan	+	+	−	−	−
H5	Oxa46	*Oxalis corniculata* L.	Tsukuba, Ibaraki, Japan	+	+	−	−	−
H5	Pas29	*Plantago asiatica*	Hitachi, Ibaraki, Japan	+	+	−	−	−
H5	St527	*Solanum tuberosum* L.	Shimabara, Nagasaki, Japan	+	+	−	−	−
H5	St528	*Solanum tuberosum* L.	Shimabara, Nagasaki, Japan	+	+	−	−	−
H5	Tre5	*Trifolium repens* L.	Kutsukake, Toyoake, Aichi, Japan	+	+	−	−	−
H6	Ost2	*Oenothera stricta* Ledeb.	Fujimi, Nagano, Japan	+	+	−	−	−
H7	Seg1	*Setaria glanea*	Fujimi, Nagano, Japan	+	+	−	−	−
H7	Seg4	*Setaria glanea*	Fujimi, Nagano, Japan	+	+	−	−	−
H8	Pc102	*Phaseolus coccineus* L.	Tsukuba, Ibaraki, Japan	−	+	−	−	−
H8	Sm6	*Solanum melongena* L.	Sakae, Toyoake, Aichi, Japan	−	+	−	−	−
RZ	Arp81	*Artemisia princeps* Pampan.	Hitachi, Ibaraki, Japan	+	−	+	−	+
RZ	Pan196	*Poa annua* L.	Tsukuba, Ibaraki, Japan	+	−	+	−	+
RZ	Pan197	*Poa annua* L.	Tsukuba, Ibaraki, Japan	+	−	+	−	+
RZ	Pc121	*Phaseolus coccineus* L.	Tsukuba, Ibaraki, Japan	+	−	+	−	+
RZ	St508	*Solanum tuberosum* L.	Minamiosumi, Kagoshima, Japan	+	−	+	−	+
RZ	St509	*Solanum tuberosum* L.	Minamiosumi, Kagoshima, Japan	+	−	+	−	+
RZ	Tre132	*Trifolium repens* L.	Tsukuba, Ibaraki, Japan	+	−	+	−	+
RZ	Van51	*Vicia angustifolia L.*	Tsukuba, Ibaraki, Japan	+	−	+	−	+

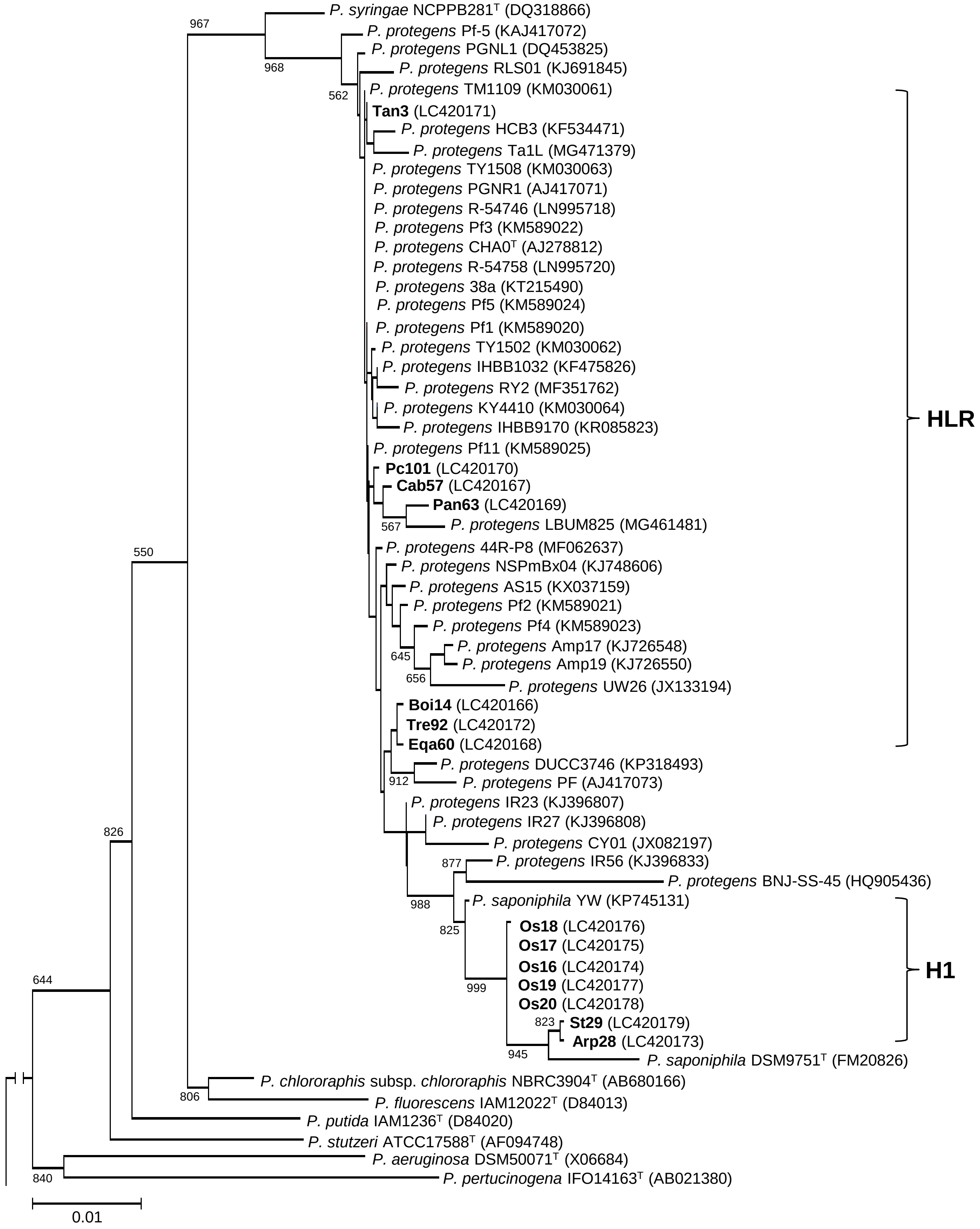

Fig. S1. Phylogenetic trees of the 16S rRNA gene sequences obtained from isolates belonging to OTU HLR and H1. As comparison, the results of a phylogenetic analysis with the 16S rRNA gene sequences from representative and related type species of genus *Pseudomonas* are indicated. *E. coli* K-12 (MG1655) was used as an out-group for the dendrogram. Bootstrap values of ≥500 (from 1,000 replicates) are indicated at the nodes.

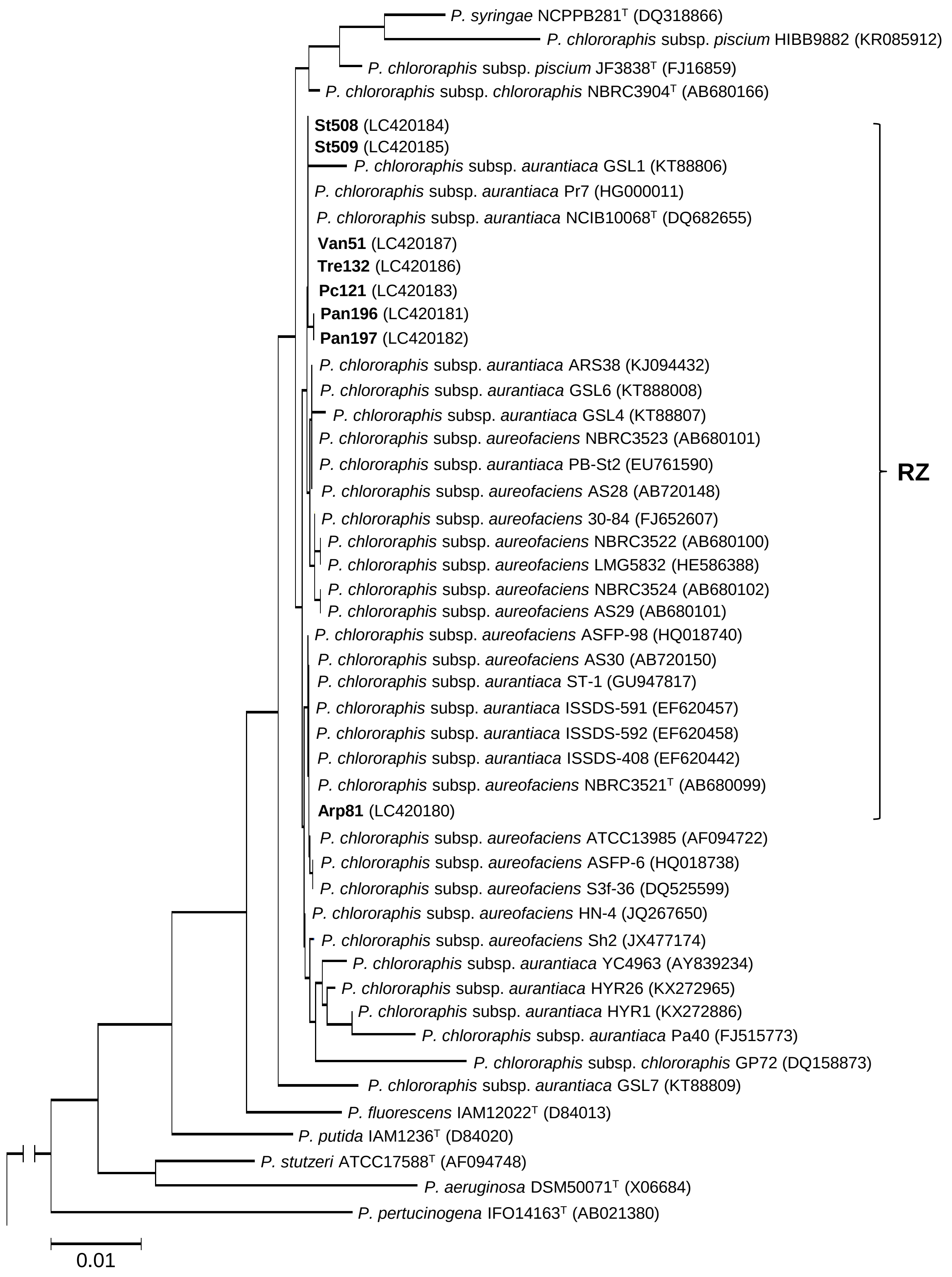

Fig. S2. Phylogenetic trees of the 16S rRNA gene sequences obtained from isolates belonging to OTU RZ. As comparison, the results of a phylogenetic analysis with the 16S rRNA gene sequences from representative and related type species of genus *Pseudomonas* are indicated. *E. coli* K-12 (MG1655) was used as an out-group for the dendrogram. Bootstrap values of ≥500 (from 1,000 replicates) are indicated at the nodes.

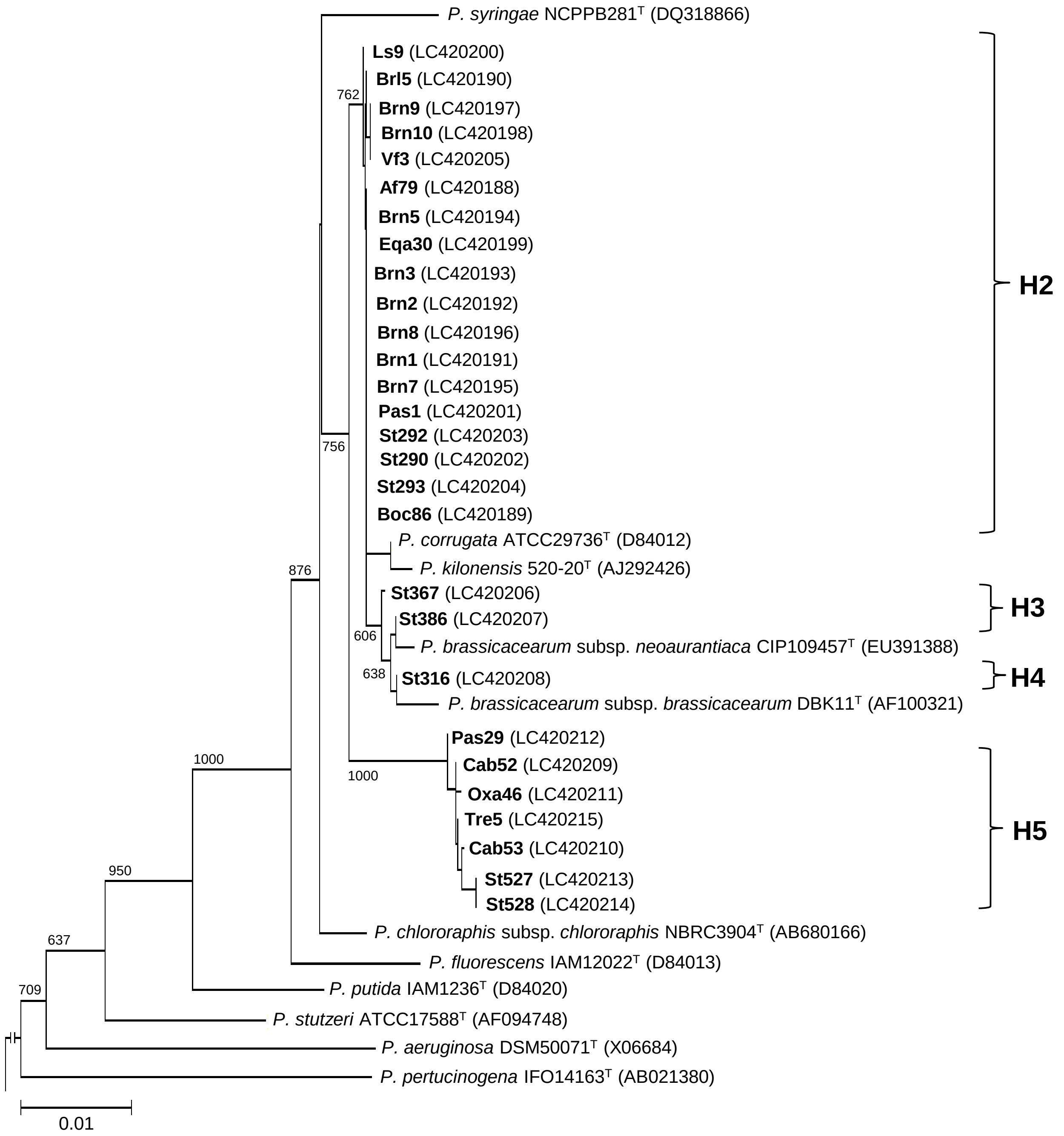

Fig. S3. Phylogenetic trees of the 16S rRNA gene sequences obtained from isolates belonging to OTU H2, H3, H4 and H5. As comparison, the results of a phylogenetic analysis with the 16S rRNA gene sequences from representative and related type species of genus *Pseudomonas* are indicated. *E. coli* K-12 (MG1655) was used as an out-group for the dendrogram. Bootstrap values of ≥500 (from 1,000 replicates) are indicated at the nodes.

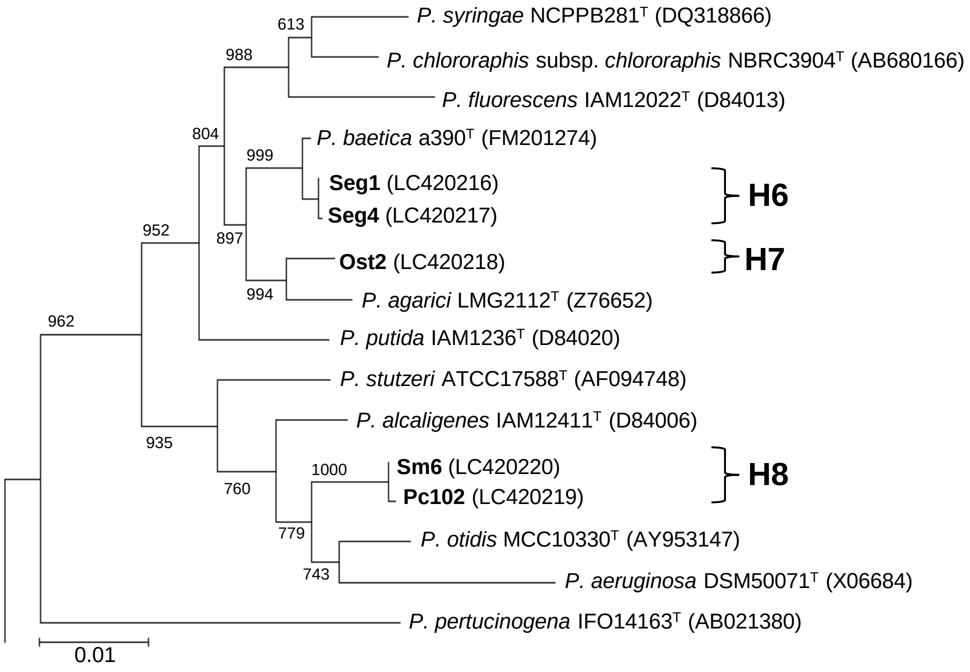

Fig. S4. Phylogenetic trees of the 16S rRNA gene sequences obtained from isolates belonging to OTU H6, H7 and H8. As comparison, the results of a phylogenetic analysis with the 16S rRNA gene sequences from representative and related type species of genus *Pseudomonas* are indicated. *E. coli* K-12 (MG1655) was used as an out-group for the dendrogram. Bootstrap values of ≥500 (from 1,000 replicates) are indicated at the nodes.
